# Cells alter their tRNA abundance to selectively regulate protein synthesis during stress conditions

**DOI:** 10.1126/scisignal.aat6409

**Published:** 2018-09-04

**Authors:** Marc Torrent, Guilhem Chalancon, Natalia S. de Groot, Arthur Wuster, M. Madan Babu

**Affiliations:** 1 Laboratory of Molecular Biology, Medical Research Council, Francis Crick Avenue, CB2 0QH Cambridge, UK; 2 Systems Biology of Infection Lab, Department of Biochemistry and Molecular Biology, Universitat Autònoma de Barcelona, 08193 Barcelona, Spain

## Abstract

Decoding the information in mRNA during protein synthesis relies on tRNA adaptors, the abundance of which can affect the decoding rate and translation efficiency. To determine whether cells alter tRNA abundance to selectively regulate protein expression, we quantified changes in the abundance of individual tRNAs at different time points in response to diverse stress conditions in *Saccharomyces cerevisiae*. We found that the tRNA pool was dynamic and rearranged in a manner that facilitated selective translation of stress-related transcripts. Through genomic analysis of multiple data sets, stochastic simulations, and experiments with designed sequences of proteins with identical amino acids but altered codon usage, we showed that changes in tRNA abundance affected protein expression independently of factors such as mRNA abundance. We suggest that cells alter their tRNA abundance to selectively affect the translation rates of specific transcripts to increase the amounts of required proteins under diverse stress conditions.

## INTRODUCTION

Translation of mRNAs into proteins is a central step during gene expression. The information in mRNA, encoded by 61 different nucleotide triplets (codons), is decoded into a protein that is composed of 20 different amino acids. In *Saccharomyces cerevisiae*, 42 nuclear-encoded transfer RNAs (tRNAs) (*1*) recognize the 61 codons and bring the corresponding amino acids to the ribosome to facilitate protein synthesis through the formation of peptide bonds. To ensure effective protein synthesis and cellular homeostasis, the anticodon demand placed by the mRNA must be balanced by the tRNA supply of the cell (*2*–*6*). An imbalance between mRNA codon usage and cognate tRNAs can affect the polypeptide elongation rate in ribosomes and induce pauses during translation that may have wide implications for homeostasis, protein quality control, and disease (*7*). These pauses may be due to changes in tRNA abundance (*8*, *9*) or modifications in certain bases (such as those in the anticodon stem) (*10*–*12*).

Despite their central role in translation, tRNAs are seen primarily as adaptor molecules with the function of ensuring correct translation (*13*). However, this view has been expanded by findings that demonstrate the tissue-specific expression of tRNA molecules (*14*) and changes in global tRNA abundance and modification during the cell cycle, development, and disease (*15*–*18*), among others (*13*, *19*–*23*). In yeast, stress-responsive genes are highly expressed but are unexpectedly enriched in codons that use rare tRNAs (*24*). We hypothesized that a dynamic tRNA pool might regulate efficient and selective translation of certain genes during stress conditions.

## RESULTS

### Quantifying changes in tRNA abundance under diverse stress conditions

**Fig. 1:**
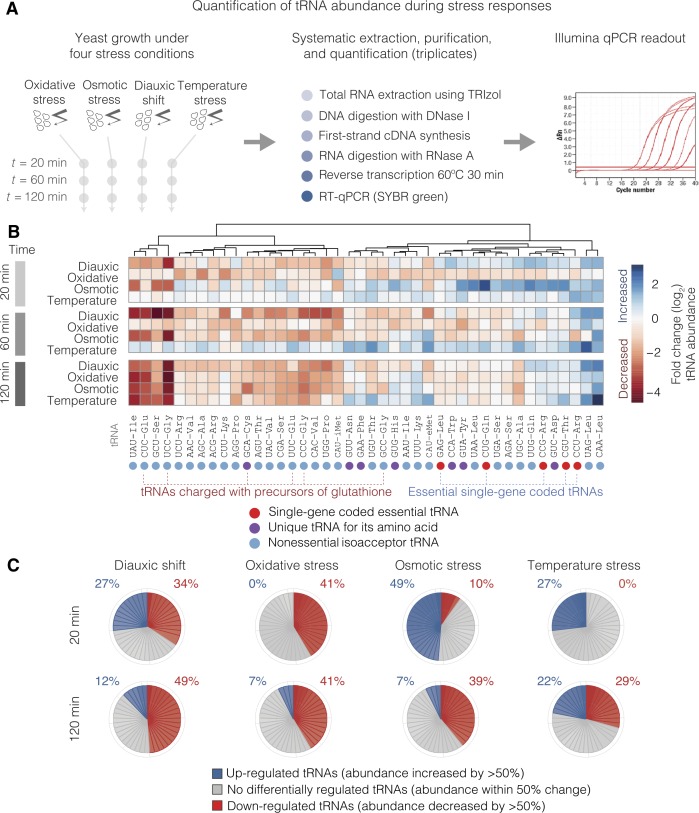
**Quantification of change in abundance of yeast tRNAs during stress.** (**A**) Yeast cells were grown in YPD (yeast extract, peptone, and dextrose) at 30°C and then challenged with four different stress conditions: (i) temperature stress by increasing incubation temperature from 30° to 37°C, (ii) osmotic stress by increasing sorbitol concentration from 0 to 1 M, (iii) oxidative stress by increasing H2O2 concentration from 0 to 0.5 mM, and (iv) diauxic shift by changing the carbon source of the media from 2% glucose to 2% ethanol. For each condition, change in abundance of individual tRNAs was measured by qPCR with respect to normal, nonstressed conditions at three different time points: 20, 60, and 120 min (all in biological triplicates). (**B**) Hierarchical clustering of tRNAs based on their relative changes in abundance over time (average fold change of *n* = 3 biological replicates). (**C**) Pie charts of the proportion of up- and down-regulated tRNAs under different stress conditions. DNase I, deoxyribonuclease I; cDNA, complementary DNA; RNase A, ribonuclease A.

We first quantified changes in abundance of each of the 42 nuclear-encoded tRNAs during adaptation to different stress conditions in the yeast *S. cerevisiae* using reverse transcription quantitative polymerase chain reaction (RT-qPCR) (Fig. 1A; table S1; fig. S1, A and B; and data file S1). There are several approaches for quantifying tRNA abundance, which include tRNA microarrays (*25*), Northern blot (*26*), and sequencing-based methods (*22*). Each of these approaches has their strengths and limitations based on various considerations such as detection sensitivity, scalability, and the ability to resolve the identity of tRNAs and discriminate cleaved tRNA fragments from mature tRNA, as well as effects of tRNA modifications. Although the efficiency of reverse transcription may vary due to nucleotide modifications in tRNAs, this approach has been validated as a reliable method to quantify mature tRNAs (*22*, *27*).

Measurement of the relative tRNA abundance profiles under four different stress conditions (oxidative stress, osmotic stress, temperature stress, and diauxic shift) at three different time points (20, 60, and 120 min) revealed that tRNA abundances changed substantially (about 2 to 5 log2 fold change; Fig. 1B) in a reproducible manner (fig. S2). Analysis of the changes in relative abundance of the individual tRNAs immediately upon stress revealed that decreasing the abundance of existing tRNA molecules (possibly through rapid degradation) could be a mechanism that changed relative tRNA abundance under different stress conditions (except under temperature stress) (Fig. 1C). The abundance of some tRNA molecules increased for all stress conditions except during oxidative stress, suggesting that active transcription could be another mechanism that regulates tRNA abundance during stress. At 120 min after stress, a higher proportion of the tRNA molecules showed decreased abundance, suggesting that repression of transcription- or degradation-based mechanisms might be a prevalent mechanism to alter tRNA abundance upon prolonged exposure to different stress conditions (Fig. 1C).

### Patterns of changes in tRNA abundance during stress

**Fig. 2:**
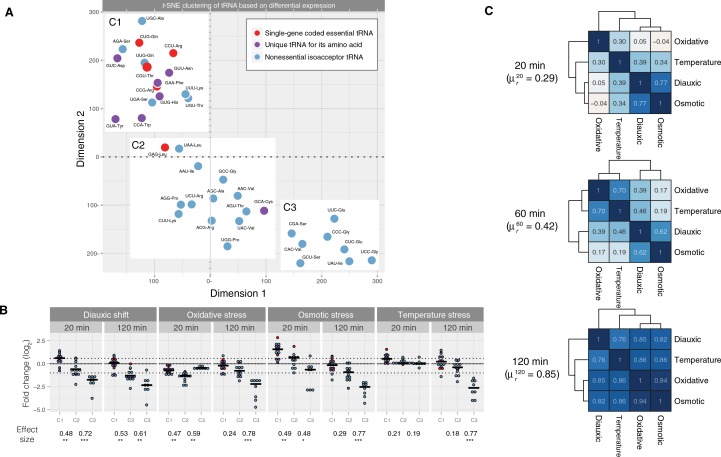
**Analysis of tRNA expression patterns.** (**A**) Multivariate analysis of the tRNA expression patterns using *t*-SNE and *K*-means clustering. Three groups of tRNAs (C1, C2, and C3) are highlighted. All tRNAs except tRNA^Leu(CAA)^, tRNA^Leu(UAG)^ (which were outliers, assignable to C1), and the initiator tRNA^Met(CAU)^ (assignable to C3) were included for the visualization. (**B**) Fold change distribution of tRNA abundance in each group (dot plots). Horizontal dashed lines indicate threshold values for up-regulation (+50%) and down-regulation (−50%). The effect sizes (rank-biserial correlation using Wendt’s criterion) of comparisons between C1-C2 and C2-C3 are indicated for each stress condition. *P* values are computed using Mann-Whitney *U* test, *P < 0.05, **P < 0.01, ***P < 0.001. (**C**) Pairwise correlation matrices showing the similarity of changes in tRNA abundance across the four stress conditions after 20, 60, and 120 min. Each cell shows the Pearson’s *r* correlation coefficient, with negative and positive values colored in shades of red and blue, respectively. μ_r_ represents global *r* values for each time point.

Using *t*-distributed stochastic neighborhood embedding [*t*-SNE (*28*)] and *K*-means clustering, we analyzed the patterns of tRNA expression across the 12 conditions and time points and found that tRNAs can be segregated into three clusters (C1, C2, and C3) (Fig. 2A). The distribution of tRNAs among the three clusters corresponded to key functional features. C1 contained four of five tRNAs that are coded by single essential genes and six of seven tRNAs that are unique acceptors of their amino acid (Fig. 2A). Almost all tRNAs in C3 (7 of 8) and more than half of the tRNAs in C1 (9 of 16) contained nonoptimal anticodons (namely, those that can form a wobble codon-anticodon pair with low affinity). In contrast, C2 was depleted of tRNAs that carry nonoptimal anticodons (4 of 14). These observations indicate that under stress conditions, the tRNA pool is rearranged to adopt a complex structure that may influence translation.

On one hand, the abundance of C1 tRNAs either increased or remained stable during stress compared to normal conditions (Fig. 2B). This result is consistent with the notion that a decrease in the abundance of these essential tRNAs would likely negatively affect cellular fitness (*21*). On the other hand, the abundance of C2 tRNAs marginally decreased or remained stable and that of C3 tRNAs decreased under all stress conditions and at all time points (Fig. 2B). Because C1 and C3 tRNAs primarily decode nonoptimal codons, we reasoned that the differences in their abundance might have a role in protein production by controlling the rate at which transcripts with nonoptimal codons are translated. The tRNAs coding for Glu, Cys, and Gly showed reduced abundance under all stress conditions (Fig. 1B). These are the three amino acids that are required for the nonribosomal synthesis of the antioxidant glutathione (GSH), which is required for adaptation to stress conditions (*29*). One possible explanation is that a feedback mechanism between the nonribosomal GSH biosynthesis pathway and ribosomal protein translation could have evolved to ensure that the precursor amino acids are available in higher abundance for GSH production under stress conditions.

From a kinetic perspective, our results showed that after 20 min of exposure to stress, changes in tRNA abundances were most different within and across stress conditions, as indicated by their average correlation, which was calculated as the average Pearson correlation on the off-diagonal elements (Fig. 2C). After 60 min of exposure, we found a better correlation (Fig. 2C); however, the correlation was the highest for prolonged stress (*t* = 120 min) (Fig. 2C). The observed changes in tRNA fold change during stress suggested a biphasic behavior during adaptation to stress (fig. S3): An immediate transient response (at 20 min) with stress-specific variations, followed by a long-term adapted response (at 120 min) in which the tRNA pool is remodeled to a similar extent under all stress conditions but altered relative to the nonstress condition.

### Genome-scale analysis of adaptation to tRNA abundance changes

**Fig. 3:**
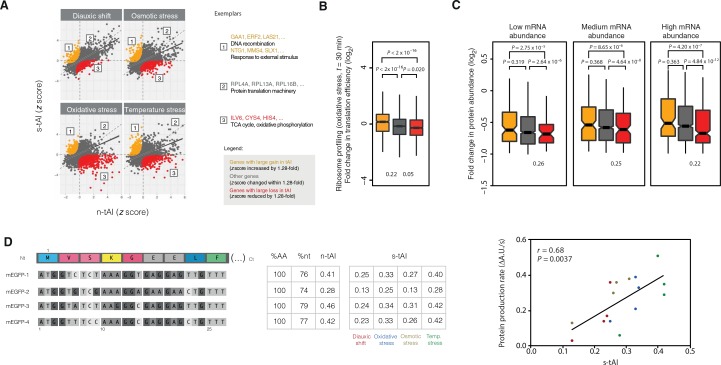
**Changes in the tAI during stress.** (**A**) n-tAI (*x* axis) and s-tAI (*y* axis) were both normalized to range between 0 and 1. Dots indicate individual genes. Orange genes have increased their *z* score of shift in rank more than 1.28 times in terms of tRNA adaptation (10% of genes that are likely to be most efficiently translated), and red genes have decreased their *z* score of shift in rank more than 1.28 times (10% of genes that are likely to be least efficiently translated) during stress compared to normal conditions. The other genes are shown in gray. Some exemplars of significant gene ontology (GO) slim enrichment are indicated in the figure (data file S4). (**B**) Ribosome profiling data during oxidative stress for a different group of genes (*n* = 4157 genes). Numbers within the box plots denote effect size. (**C**) Increase in protein abundance during oxidative stress for the different group of genes. To control for mRNA abundance, three plots are shown for low (*n* = 1386 genes), medium (*n* = 1386 genes), and high (*n* = 1385 genes) mRNA abundance (tertile classification). (**D**) Experimentally measured protein production rates of the four variants of mEGFPs calculated as an increase in fluorescence per time (ΔA.U./s) under different stress conditions (average of *n* = 3 biological replicates). Amino acid similarity (%AA), nucleotide similarity (%nt), n-tAI, and s-tAI values are shown. mEGFP protein production rates for the four variants under each of the four stress conditions (16 measurements) plotted against s-tAI. Individual *r* values for each stress condition are: 0.66 (diauxic shift), 0.77 (oxidative stress), 0.98 (osmotic stress), and 0.77 (temperature stress). A.U., arbitrary units; Nt, N terminus; Ct, C terminus; TCA, tricarboxylic acid.

Given that the tRNA abundance influences the decoding rate of codons during translation (*7*, *30*), our observations implied that the rate of protein synthesis of individual genes might be selectively affected during stress. To investigate this notion, we first measured the extent to which the codon usage of all yeast genes was adapted to the tRNA abundance under normal and each of the four stress conditions. This was quantified by computing the tRNA adaptation index under normal conditions (n-tAI) and a newly developed metric, stress-adjusted tAI for each of the four stress conditions (s-tAI) (Fig. 3A, and data files S2 and S3), using the experimental measurements described above. For each stress condition, we identified genes that were better adapted, not affected, and less adapted to the experimentally measured tRNA pool by obtaining a *z* score of the rank change of their n-tAI and s-tAI values [Fig. 3A (orange, gray, and red data points) and data file S3].

We found that the genes with better codon adaptation to the tRNA pool under the different stress conditions were specifically enriched for functions related to external stimulus responses (Fig. 3A and fig. S4). Genes whose codon adaptation was not substantially altered were enriched in ribosomal proteins and translation machinery. Finally, the ones whose codon usage patterns were less well adapted were enriched in anabolic functions such as amino acid and lipid biosynthesis enzymes and tricarboxylic acid cycle (Fig. 3A, fig. S4, and data file S4). Although this observation suggested that the translation efficiency (TE) of certain genes might be selectively altered, we still observed a global positive correlation between tRNA supply and codon demand under all stress conditions, suggesting that overall translation was unlikely to be substantially compromised (fig. S5, A and B).

### Alteration in translation rate and protein abundance during stress

To assess whether the genes that were identified as better adapted to the tRNA pool during stress, based on the changes in s-tAI ranks (Fig. 3A), showed a gain in TE, we analyzed experimentally derived ribosome footprinting data measured under oxidative stress (*31*, *32*). The median log2 TE (or the amount of footprint normalized to mRNA abundance) of genes with better codon adaptation under oxidative stress was substantially higher compared to that of genes whose adaptation remains unaffected (Fig. 3B). Genes that were less well adapted had a lower median log2 TE compared to genes whose adaptation remained stable during oxidative stress (Fig. 3B).

Furthermore, investigation of protein abundance data during oxidative stress, as measured using confocal microscopy (*33*), revealed that genes that were better adapted to stress showed a significant increase in protein abundance compared to those that were less well adapted, although their mRNA abundance were comparable (Fig. 3C). These observations collectively suggest that changes in tRNA abundances might be an independent mechanism that can selectively increase the abundance of proteins encoded by certain transcripts that are better adapted (in terms of their codon usage) to the tRNA pool under stress condition.

### Measuring protein production rates of designed sequences during stress

To experimentally determine whether changes in the tRNA pool can influence protein production rates, we designed monomeric enhanced green fluorescent protein (mEGFP) variants with identical amino acid sequence but different codon usage. Because most changes in the tRNA abundance occur immediately after stress (*t* = 20 min), we used the tRNA fold change data in this time window to design mEGFP variants. We examined each codon in the wild-type mEGFP sequence and substituted it with a synonymous codon if the fold change of the relevant isoacceptor tRNA increased more (or decreased less) than its native cognate tRNA (Fig. 3D and table S2). If the isoacceptor tRNA could recognize more than one codon, then the new codon was picked at random.

Because mRNA structure can affect translation (*34*, *35*), we ensured that the designed transcripts did not contain any unusual secondary structure and had comparable free energies of folding using the ViennaRNA package (fig. S6). To avoid variations in mRNA abundance due to position effect, or due to plasmid copy number, we integrated each mEGFP variant in the TRP1 region of the yeast genome under a GAL1 promoter and obtained four distinct yeast strains that express the mEGFP gene (fig. S7). After confirming that all strains showed similar mEGFP mRNA levels (fig. S8), we measured the increase in fluorescence over time (as a proxy for protein production rate) of each strain subjected to the four stress conditions. We observed a positive relationship between the computed s-tAI values for the designed transcripts and the respective protein production rates (jointly and independently) for the different stress conditions (Fig. 3D). Although the relationship between these variables may be nonlinear, it was best modeled by a linear fit. This suggests that the changes in s-tAI affect the protein production rates of different mEGFP variants.

Together with the observations from the genomic analysis (Fig. 3, A to C), these results suggest that changes in tRNA abundance in response to stress might globally influence the proteome by selectively altering the protein production rates of transcripts under different stress conditions. Thus, transcripts with codons that are better adapted to the tRNA pool under a given stress condition are likely to be selectively translated with higher efficiency, leading to an increased abundance even after controlling for mRNA abundance and structure. Our data also show that the TE of the same transcript may be substantially altered when the tRNA pool is altered during stress, although transcript abundance remains the same.

### Inferring the impact of s-tAI on protein production during stress

**Fig. 4:**
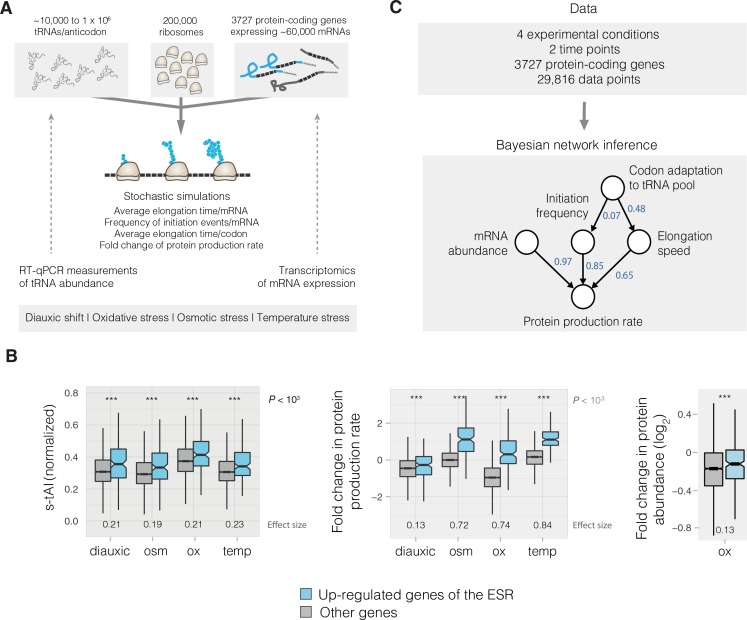
**Simulation of protein translation based on the experimentally measured changes in tRNA.** (**A**) Simultaneous simulation of protein production rates for 3727 yeast genes. (**B**) Distribution of s-tAI values and fold change in protein production rates for the ESR up-regulated genes (blue) compared to the other genes (gray). Fold change in protein abundance (right) in oxidative stress based on confocal microscopy. (**C**) Bayesian network inference. The explanatory variables used to study protein production rate fold change include the following: variation in mRNA abundance, tAI, initiation frequency, and elongation speed. In total, 29,816 observations were pooled from simulations of the four stress conditions. The numbers on the edges in the network represent the magnitude of the link strength. *P* values are computed using Mann Whitney *U* test, *P < 0.05, **P < 0.01, ***P < 0.001. Numbers within the box plots denote effect size.

To characterize the possible impact of tRNA abundance changes on the *S. cerevisiae* proteome during stress, we used a stochastic model developed by Shah and co-workers (*36*) and simulated translation based on the experimentally measured changes in mRNA (*37*) and tRNA abundance (Fig. 4A and fig. S9). Consistent with our observation that stress-related genes showed better adaptation to the tRNA pool and increased protein abundance (Fig. 3C), we observed that the genes that are specifically up-regulated transcriptionally during the environmental stress response (ESR) showed a higher s-tAI and increased protein production rate in our simulations compared to other genes (Fig. 4B, left and middle). Thus, the combined effects of increased mRNA abundance and a higher s-tAI act in favor of increasing protein production rates of ESR up-regulated genes during stress. In line with the simulation results, the experimentally observed change in protein abundance (with respect to nonstressed condition) is significantly higher for the ESR genes during oxidative stress compared to the other genes (Fig. 4B, right).

We observed that a predicted increase in protein production rate was coupled with a slight decrease in overall steady-state protein abundance during oxidative stress (Fig. 4B). This effect may be because translation is globally reduced during oxidative stress (*37*), and most changes in tRNA abundance are due to reduced abundance rather than increased abundance (Fig. 1C). Despite the global reduction in translation, better adaptation of stress genes to the tRNA pool suggested that they were relatively rapidly translated compared to the other genes. The decrease in overall protein abundance could also be due to different gene sets used for the simulations and for which abundance measurements were available. Finally, the difference may also be explained by an increased degradation rate of proteins under stress, which might skew the overall protein abundance despite an increase in protein production rate.

To quantify the relative contribution of different factors such as changes in transcript abundance and codon adaptation to tRNA pools on the protein production rate, we performed an unbiased analysis using Bayesian statistics on the data obtained from the simulation. The most likely model that best explains the data showed that changes in protein abundance are highly dependent on changes in tRNA abundance, which contribute substantially to protein production rates, and are themselves explained by changes in tAI of the genes (Fig. 4C). In line with previous findings, regulation of translation initiation and changes in mRNA abundance also greatly influence protein production rate (*34*, *36*, *37*). Consistent with the observations from the experimental data (Fig. 3, C and D), we also found that changes in mRNA abundance were independent from tRNA-related effects (Fig. 4C), namely, changes in mRNA abundance and tRNA abundance are two distinct levers that regulate protein production. This suggests that changes in tRNA abundance can alter the relative translation rates of transcripts throughout the transcriptome and selectively influence the production rates, and hence abundances, of proteins during stress. Therefore, codon adaptation to the tRNA pool is an independent mechanism that can fine-tune protein abundance and complements other mechanisms such as transcriptional regulation, transcript degradation, and regulation of translation initiation.

## DISCUSSION

**Fig. 5:**
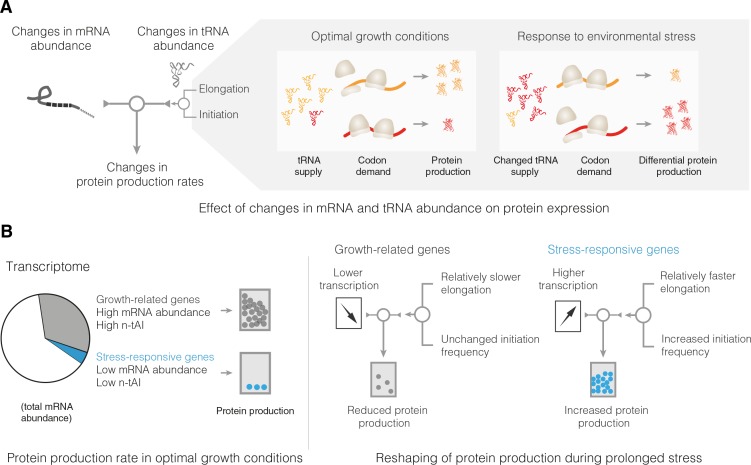
**A model for how changes in the tRNA abundance could selectively influence protein production rates of specific transcripts.** (**A**) Under prolonged stress, the production of proteins is reshaped as a result of changes in the abundance of mRNAs and tRNAs. In this scenario, there is a balance between codon demand and tRNA supply, whereby adaptation to the tRNA pool may result in higher levels of protein production. (**B**) Under optimal growth conditions, the transcriptome consists of highly abundant mRNAs coding for growth-related genes whose codon usage is adapted to tRNA abundance under normal conditions and whose proteins are produced at a high rate and abundance (gray). Another part of the transcriptome consists of lower abundance mRNAs for stress-responsive genes whose codons are less adapted to tRNA abundance under normal conditions and whose proteins are produced at basal or low levels (blue). After prolonged stress, the tRNA pool is significantly altered. Growth-related genes tend to have fewer transcripts and show relatively slower elongation due to reduced codon adaptation to the new tRNA pool, resulting in decreased protein production. Stress-responsive genes tend to have more transcripts whose elongation is also globally slower than in normal conditions but relatively faster compared to the other genes because of better codon adaptation to the new tRNA pool, resulting in an overall increase in protein production.

In response to stress, certain proteins need to be synthesized rapidly and in higher abundance to ensure that cells adapt to new conditions. However, immediately after experiencing stress, both new and previously transcribed mRNAs may be present in comparable abundance for translation by the ribosome (*38*). In addition to other mechanisms such as sequestration of mRNA into P bodies and stress granules, selective translation initiation, tRNA modification, and selective degradation of transcripts based on codon usage (*39*–*44*), we suggest that changes in the tRNA pool ensure that newly synthesized stress-related transcripts are selectively translated with higher efficiency by the ribosome compared to the already present mRNA, thereby leading to a selective increase in the abundances of the required proteins (Fig. 5, A and B). The different mechanisms such as changes in tRNA abundance and nucleotide modification may act in concert to affect translation. For instance, we observed an increase in tRNA^Leu(CAA)^ that, together with an increase in the proportion of tRNA^Leu(CAA)^ containing m5C at the wobble position, may cause a significant translational bias toward TTG-enriched proteins.

The findings presented here open up a number of questions, such as how the abundances of individual tRNAs are regulated. Genome-wide screens may aid the discovery of specific transcriptional regulators (*45*) and nucleases (*46*) that may have a role in tRNA synthesis and degradation, respectively. Furthermore, several factors that regulate the tRNA status such as aminoacylation (*47*), nucleocytoplasmic localization (*48*), and nucleotide modifications (*10*, *49*–*52*) can fine tune the activity of individual tRNAs and affect protein production rates of specific transcripts. Despite these considerations, our data reveal an important role for tRNA in selectively regulating protein production rates, whereby changes in tRNA abundance results in the altered TE for the same mRNA (Fig. 5, A and B). Thus, changes in tRNA abundance should be added as an important layer of regulation in the central dogma of gene expression.

## MATERIALS AND METHODS

### Yeast strains and culture

All strains used in this work were based on Y03157 (BY4741; Mat α; his3Δ1; leu2Δ0; met15Δ0; ura3Δ0; YBR020w::kanMX4) obtained from Euroscarf deletion collection. Yeast strains were grown in YPD medium at 30°C (nonstress control). For stress conditions, changes were introduced as follows: (i) temperature was increased from 30° to 37°C (temperature stress); (ii) cells were gently centrifuged at 3000*g* for 2 min, and the medium was replaced by YPD containing 0.5 mM H2O2 (oxidative stress); (iii) cells were gently centrifuged at 3000*g* for 2 min, and the medium was replaced by YPD containing 1 M sorbitol (osmotic stress); and (iv) cells were gently centrifuged at 3000*g* for 2 min, and the medium was replaced by YPD containing 2% ethanol instead of 2% glucose as carbon source (diauxic shift). Cultures were followed for 120 min, and aliquots for further analysis were obtained at 0, 20, 60, and 120 min after stress.

### RNA extraction

RNA was extracted from yeast cells using TRIzol phenol-chloroform extraction. Briefly, 5 ml of yeast culture were centrifuged at 3000*g* for 2 min. Yeast cells were resuspended in 150 μl of lysis buffer (0.1 M lithium acetate and 0.5% SDS) and heated at 70°C for 5 min. After that, 450 μl of TRIzol LS reagent was added and mixed for 15 s. Then, 150 μl of chloroform was added, mixed for 15 s, and incubated at room temperature for 5 min. Samples were centrifuged at 12,000*g* during 30 min, and the aqueous phase was obtained. RNA was then precipitated with 450 μl of isopropanol. The RNA pellet was recovered by centrifugation at 10,000*g* for 20 min and then cleaned twice with 75% ethanol. Finally, RNA was dissolved in 25 μl of sterile RNase-free water (Life Technologies) and immediately used for cDNA synthesis. The samples were quantified using a NanoDrop ND-1000 (Thermo Fisher Scientific). All samples had an A260/A230 ratio of >2 and A260/A280 ratio of >2.

### cDNA synthesis

To ensure that the isolated RNA was free from DNA, samples were incubated with RNase-free DNase I (Sigma-Aldrich) for 30 min according to the manufacturer’s instructions. The reaction was stopped by adding 5 mM EDTA (Sigma-Aldrich) and heating the sample at 70°C for 10 min. Then, 1 μg of RNA was reverse-transcribed using the RevertAid First Strand cDNA Synthesis Kit (Thermo Fisher Scientific). To minimize the effect of secondary structure, the reaction was conducted at 60°C (instead of 45°C) and extended for 30 min (instead of 15 min) to reduce the effect of transcription pauses due to tRNA modifications.

### Quantification of individual tRNAs

Individual tRNAs were quantified by qPCR on an ECO Real-Time qPCR thermocycler (Illumina). One microliter of a 1:1000 dilution of cDNA from the reverse transcription reaction and 175 μM of the corresponding primers were used for the PCR reaction. Primers were designed using Primer-BLAST (https://ncbi.nlm.nih.gov/tools/primer-blast/) to ensure that each sequence is specific for the template and does not hybridize with any other region of the *S. cerevisiae* genome (table S1 and fig. S1, A and B). The PCR reaction was conducted using the SYBR Green PCR Master Mix (Life Technologies), and the results obtained were processed using the ΔΔ*C*t method. The geometric mean of four housekeeping genes (*ALG9*, *TAF10*, *TFC1*, and *UBC6*) was used as reference (*53*, *54*). No bias was observed in qPCR efficiency for the 42 primer pairs designed (table S1 and fig. S1, A and B). All quantifications were done using biological triplicates.

### Design of mEGFP genes

Four mEGFP sequences were designed to test the effect of codon usage and altered tRNA abundance on protein translation. The sequences were designed using the Visual Gene Developer software (*55*) using the following criteria: All codons were substituted by the most up-regulated codon under the particular stress condition. In those cases where the same tRNA translated two codons, we randomly chose the codon. We also tested that all transcripts derived from our genes have similar RNA folding energy (fig. S6). Modified mEGFP sequences are in table S2.

### Construction of GFP genes and cloning into yeast

A pMA synthetic vector carrying a multicloning site flanked by 60– base pair regions that is homologous to TRP1 was purchased from GeneArt (Life Technologies). *TRP1* gene was chosen as the position for insertion due to its close proximity to the centromere and the high expression levels of genes in this region; these properties will likely minimize the frequency of gene silencing and will ensure a steady-state expression level through several generations. All GFP versions were synthesized de novo (GeneArt, Life Technologies) and inserted in the pMA vector between pGal1 and TEF terminator using the In-Fusion HD Cloning Kit (Clontech). A plasmid scheme is displayed in fig. S7. The constructs were propagated in *Escherichia coli* and purified using a QIAprep kit (Qiagen). The insertion sequence was amplified by PCR, and the product was purified by agarose gel electrophoresis using a MinElute kit (Qiagen). Finally, the gene was introduced in the *S. cerevisiae* genome by homologous recombination using the lithium acetate protocol (*56*). To ensure that the gene was inserted in the correct position, it was amplified using PCR after recombination and sequenced.

### mRNA expression levels

Fresh cells (5 ml) were grown with 1% galactose, centrifuged and suspended with 1 ml of 0.2 M lithium acetate and 1% SDS solution, and boiled at 70°C for 5 min. Then, RNA was extracted as detailed before. qPCR was performed using the SYBR Green PCR Master Mix (Life Technologies) as described before using the primers listed in table S3.

### Fluorescence measurements

*S. cerevisiae* cells expressing the four modified mEGFP constructs were grown in a plate reader (Tecan) monitoring both mEGFP fluorescence and cell growth [optical density at 600 nm (OD600)] over time. Fluorescence measurements were normalized by cell number (measured as fluorescence/OD600) and corrected for autofluorescence. We also measured the transcript abundance for the different sequences and did not observe any difference in terms of the mRNA expression levels (fig. S8). The slope of normalized fluorescence over 3 hours (▲A.U./s) was taken as a measure of protein production.

### Computational analysis and stochastic simulations

Details on computational analysis, simulation, and statistical evaluation of the data presented can be found in the Supplementary Materials.

## Supplementary Material

Cells alter their tRNA abundance to selectively regulate protein synthesis during stress conditions

Cells alter their tRNA abundance to selectively regulate protein synthesis during stress conditions

Cells alter their tRNA abundance to selectively regulate protein synthesis during stress conditions

Cells alter their tRNA abundance to selectively regulate protein synthesis during stress conditions

Cells alter their tRNA abundance to selectively regulate protein synthesis during stress conditions

## SUPPLEMENTARY MATERIALS

www.sciencesignaling.org/cgi/content/full/11/546/eaat6409/DC1

Materials and Methods

Fig. S1. PCR efficiency, GC content, and sequence identity of PCR primers.

Fig. S2. Noise associated with each tRNA measurement for the different stress conditions.

Fig. S3. Biphasic response of tRNA abundance change upon exposure to stress.

Fig. S4. Functional enrichment of genes whose codons are better or less well adapted to stress conditions.

Fig. S5. Balance between anticodon supply and demand in stress conditions.

Fig. S6. Computed mRNA folding energy of the four different mEGFP variants.

Fig. S7. Plasmid map used for integrating the mEGFP variants into the yeast genome.

Fig. S8. mRNA abundance measurement for the mEGFP variants under stress conditions.

Fig. S9. Stress-induced fold changes in protein production rates during simulation.

Table S1. Oligos used for tRNA quantification.

Table S2. mEGFP variant sequences.

Table S3. Oligos used for mRNA quantification.

Data file S1. tRNA relative abundance during the different stress conditions.

Data file S2. Codon adaptiveness values for the different stress conditions.

Data file S3. n-tAI and s-tAI values of genes for the different stress conditions.

Data file S4. Summary of GO term analysis.

References (*57*–*69*)
