# Supplementary material for: Cells alter their tRNA abundance to selectively regulate protein synthesis during stress conditions
Source: Sci Signal. 2018 Sep 4;11(546):eaat6409. doi: 10.1126/scisignal.aat6409 (PMC6130803; doi:10.1126/scisignal.aat6409)
Supplement: Cells alter their tRNA abundance to selectively regulate protein synthesis during stress conditions [file SS-11-eaat6409-s1.pdf]

Science Signaling

AAAS

www.sciencesignaling.org/cgi/content/full/11/546/eaat6409/DC1

Supplementary Materials for

Cells alter their tRNA abundance to selectively regulate protein synthesis during stress conditions

Marc Torrent*, Guilhem Chalancon, Natalia S. de Groot, Arthur Wuster, M. Madan Babu*

*Corresponding author. Email: marc.torrent@uab.cat (M.T.); madanm@mrc-lmb.cam.ac.uk (M.M.B.)

Published 4 September 2018, *Sci. Signal.* **11**, eaat6409 (2018)
DOI: 10.1126/scisignal.aat6409

The PDF file includes:

Materials and Methods
Fig. S1. PCR efficiency, GC content, and sequence identity of PCR primers.
Fig. S2. Noise associated with each tRNA measurement for the different stress conditions.
Fig. S3. Biphasic response of tRNA abundance change upon exposure to stress.
Fig. S4. Functional enrichment of genes whose codons are better or less well adapted to stress conditions.
Fig. S5. Balance between anticodon supply and demand in stress conditions.
Fig. S6. Computed mRNA folding energy of the four different mEGFP variants.
Fig. S7. Plasmid map used for integrating the mEGFP variants into the yeast genome.
Fig. S8. mRNA abundance measurement for the mEGFP variants under stress conditions.
Fig. S9. Stress-induced fold changes in protein production rates during simulation.
Table S1. Oligos used for tRNA quantification.
Table S2. mEGFP variant sequences.
Table S3. Oligos used for mRNA quantification.
References (*57*–*69*)

Other Supplementary Material for this manuscript includes the following:

(available at www.sciencesignaling.org/cgi/content/full/11/546/eaat6409/DC1)

Data file S1 (Microsoft Excel format). tRNA relative abundance during the different stress conditions.
Data file S2 (Microsoft Excel format). Codon adaptiveness values for the different stress conditions.
Data file S3 (Microsoft Excel format). n-tAI and s-tAI values of genes for the different stress conditions.
Data file S4 (Microsoft Excel format). Summary of GO term analysis.

MATERIALS AND METHODS

Estimation of mRNA abundance during stress

To estimate mRNA abundance of transcripts in the 9 conditions tested, we integrated sequencing data from Ingolia et al. (*57*) for normal conditions and microarray data from Gasch et al. (*37*) In the microarray dataset, we selected the stress conditions that closely matched the experimental conditions in which changes of tRNA abundance were measured. To quantify the mRNA abundance *a* of individual genes *g* during the stress *s* –noted $a_g^{(s)}$-, we first used an estimate of the total number of expressed mRNAs (RPKM) in exponentially growing yeast cells, T_{mRNA} = 60,000 mRNAs (*58*). Based on these numbers, we derived the abundance of mRNAs in normal conditions, $a_g^{(n)}$:

$$a_g^{(n)} = \frac{RPKM^{(n)}(g)}{\sum RPKM^{(n)}} \times T_{mRNA} \qquad (1)$$

The mRNA abundance of genes in stress conditions, $a_g^{(s)}$, was deduced from the product of $a^{(n)}$ and the mRNA fold change (in abundance) during stress. We then restricted our analysis to the 3,794 mRNAs that were included in the stochastic simulation of protein translation (*36*).

Obtaining codon usage and anticodon demand

U_i, the total number of occurrences of codon *i* was computed by summing up the occurrences of codon *i* in each gene (noted c_{ig}), weighted by the mRNA abundance a_g.

$$U_i = \sum_{g=1}^{G} a_g c_{ig} \qquad (2)$$

Following the work by Pechmann et al. (*4*), we defined codon usage cu_i as a relative estimate of how often each codon is translated, in a given a transcriptome *G*. We computed cu_i by rescaling U_i to have a maximum of 1:

$$cu_i = U_i / U_{max} \qquad (3)$$

Codon usage was computed in normal conditions using $a_g^{(n)}$ and in different stress conditions using the corresponding transcript abundance $a_g^{(s)}$.

The anticodon demand for anticodon *j* in condition *s* – noted $D_j^{(s)}$ – was computed by aggregating the codon usages of all the codons recognized by *j*. For instance, in the case of $tRNA^{Val(AAC)}$:

$$D_{AAC}^{(s)} = cu_{gtc}^{(s)}(g) + cu_{gtt}^{(s)}(g) \qquad (4)$$

Obtaining anticodon supply

Data on estimated tRNA abundance were used to define the supply of each 41 elongating anticodon. For this, we used a quantification by Waldron and Lacroute (*59*) who measured that normal yeast cells contain about $T_{tRNA} = 3.3\text{x}10^6$ tRNA molecules in exponential growth. In normal conditions, tRNA supply is proportional to tRNA Gene Copy Numbers (tGCN) (*13, 60*). We therefore used tGCN to estimate the fraction of T_{tRNA} that each tRNA represents.

$$S_j^{(n)} = \frac{tGCN^{(n)}(j)}{\sum tGCN^{(n)}} \times T_{tRNA} \qquad (5)$$

To estimate the supply of tRNAs in different stress conditions, we multiplied the abundance in normal conditions by the observed fold-change that we experimentally determined during the different stress conditions. For instance, the abundance of $tRNA^{Lys(CUU)}$ in normal conditions would be:

$$S_{CUU}^{(n)} = \frac{14}{273} \times 3.3 \times 10^6 \approx 169{,}230\ tRNAs$$

For a fold change of 0.2 for a tRNA in oxidative stress (20 min), the anticodon supply was computed as 20% of the abundance in normal condition by 0.2:

$$S_{CUU}^{(ox)} \approx 33{,}850\ tRNAs$$

Defining stress-adjusted tRNA adaptation indices

The tRNA Gene Copy Number (tGCN) indicates the number of nuclear tRNA genes of each tRNA species, namely for each unique anticodon. Because it correlates with the molecular abundance of tRNAs in exponentially growing cells, tGCN is considered to be a good predictor of tRNA abundance (in optimal conditions) for various model organisms (*6, 61*). One advantage of tGCN is that it is relatively easily computable in well-sequenced genomes with the use of tRNA gene predictors such as tRNAscan-SE (*62*). Dos Reis *et al.* thus used tGCN as a proxy for tRNA abundance (*63*), making their method usable with species for which the annotation of tRNA genes has been made but a quantification of tRNA abundance is lacking.

To re-compute tRNA adaptation index (tAI) calibrated for various stress conditions, we first computed stress adjusted tGCN values, noted $tGCN_{(s)}$, by multiplying the known $tGCN_{(n)}$ by their fold changes $FC_{(s)}$ measured during stress (**Data File S2**). The CAU anticodon (which recognises the ATG codons) required specific attention, because yeast possesses two types of $tRNA^{Met(CAU)}$ that are involved in the recognition of the AUG start codon, $tRNA^{Met.i(CAU)}$, or involved in the decoding of AUGs during elongation, $tRNA^{Met.e(CAU)}$ (*64*). These two types of $tRNA^{Met(CAU)}$ differ in their sequence, which we used to specifically amplify tRNAs of both types. However, because the algorithm to simulate translation from Shah *et al.* (*36*) cannot distinguish between these two types of tRNAs, we computed a proxy for $tGCN_{(s)}^{CAU}$ using the following weighted sum:

$$tGCN_{CAU}^{(s)} = tGCN_{CAU.i}^{(n)} \times FC_{CAU.i}^{(s)} + tGCN_{CAU.e}^{(n)} \times FC_{CAU.e}^{(s)} \qquad (6)$$

After calculating $tGCN^{(s)}$, we estimate the relative adaptiveness of each codon, noted w_i, to any given tRNA pool, using the metric described by dos Reis *et al.* (*65*), as a preceding step for tAI calculation. This metric accounts for the two crucial factors that influence the average elongation time of a codon: (i) the stability of the anticodon-codon pair (which relates to Crick's wobble rules), and (ii) the concentration of the considered tRNA. Most adapted codons combine low dissociation constant and high tRNA concentration (*63, 65*).

In the calculation of w_i, the first constraint, noted as s_{ij}, describes the stability of the pairing between the 5'-anticodon nucleotide in *j* and the 3' codon nucleotide in *i*. Non-cognate anticodon-codon pairs have a $0 < s < 1$, and non-permitted pairs have a score of 1. With the assumption that tRNA usage should be maximal in highly expressed genes, dos Reis *et al.* (*63*) optimized the s_{ij} values of wobbling codon-anticodon pairs using the Nelder-Mead algorithm. The W-score of a codon is then computed as the weighted sum of the selection constraint and tGCN for all the n_i triplets of nucleotides that, according to Crick's wobble rules, can pair with the codon *i* (including non-existing anticodons *j*, for which tGCN=0):

$$W_i = \sum_{j=1}^{n_i} (1 - s_{ij}) tGCN(j) \qquad (7)$$

The W-scores are then normalized, thereby defining the relative adaptiveness *w*. This scaling allows systematically comparing the adaptiveness of different codons. Typically, a value of $w_i = 1$ indicates that the codon *i* is the most adapted codon to the tRNA pool, and a value close to 0 indicates that the codon is very poorly adapted, and will likely take a long time to elongate.

$$w_i = \begin{cases} W_i / W_{max} & \text{if } W_i \neq 0 \\ w_{mean} & \end{cases} \qquad (8)$$

In their original paper, dos Reis *et al.* (*63*) normalized *W* by the maximal adaptiveness W_{max}. We found that this approach is particularly sensitive to outliers, in the cases where W_{max} is far higher than other *W* values. To circumvent this, we used a more robust normalizing factor, W_N computed by the geometric mean of the 3 highest *W* values:

$$\begin{cases} W_N = \sqrt[3]{\prod_{i \in top3} W_i} \\ w_i = \min(1, \frac{W_i}{W_N}) \end{cases} \qquad (9)$$

The use of W_N allows reducing the impact on a single (distant) data point on the normalization. We recomputed *w* for all 64 codons under the normal conditions and found a $r > 0.99$ correlation with dos Reis *et al.* (*63*) measurements.

Based on the quantification of tRNA abundance during stress, we computed stress-adjusted relative adaptiveness scores $w_i^{(s)}$ for the four stress responses studied at two time points (t=20 min and t=120 min); (**Data File S3**). For this, we updated Eq. 7 by replacing $tGCN_{(n)}$ by the $tGCN_{(s)}$ defined before. Therefore, $w_{(s)}$ scores account for changes in tRNA concentration, and describe how efficiently a given codon is elongated given the wobbling constraints and the abundance of its tRNA.

Stress-adjusted tRNA Adaptation Indexes (denoted s-tAI$_{(s)}$), are straightforward to compute once $w^{(s)}$ scores are available. For a given gene g exposed to a stress condition s, $s\text{-}tAI_{(s)}(g)$ is the geometric mean of the relative adaptiveness of all the codons of g in that condition.

$$s - tAI_{(s)}(g) = \left(\prod_{k=1}^{n_{codon(g)}} w_k^{(s)}\right)^{1/n_{codon(g)}} \qquad (10)$$

Where $n_{codon}(g)$ is the number of codon of gene g excluding the stop codon.

Stochastic simulation of protein translation

We used the algorithm and code from Shah *et al.* (*36*) to simulate the effect of change in tRNA abundance during stress on the translation dynamics of 3,794 yeast transcripts for which initiation probabilities had been estimated (*57*). The model considers the diffusion of ribosomes and tRNA molecules in the cell, assuming a spherical volume V = 4.2x10^{-17} m^3 that corresponds to the average cytosolic volumes of yeast cells (*66*). Every gene with ribosome density information (*57*) was included in the simulation with quantified mRNA expression levels, yielding a total of 3,794 genes, and summing up to 60,000 individual mRNA molecules (mRNA abundance ranging from 1 to 1,254 mRNA copies/gene), and competing for the same resources. Translation initiation and elongation events were monitored at each time point of the simulation for 1500 s (≈ 25 min) after a "heating" period of 1000 s. Shah *et al.* (*36*) defined initiation rates for individual genes as:

$$\rho_i = p_i \frac{R^f}{\tau_r N_r} \qquad (11)$$

where R^f represents the total number of free ribosomes, τ_r the characteristic time of diffusion of ribosomes, and N_r the number of available discrete positions for ribosomes in the cell volume. The term p_i denotes the probability that initiation will start, knowing that a ribosome has diffused to the mRNA.

Elongation rates were defined for individual genes as:

$$\varepsilon_i = \frac{1}{\sum_{j=1}^{61} x_j c_j} \qquad (12)$$

where x_j denotes the number of occurrences of codon j in the CDS of the gene i, and c_j defines the time of elongation of that type of codon, given the quantity of free cognate tRNAs recognizing j, the wobble parameter associated to the anticodon-codon pair w_j, the characteristic time of diffusion of tRNAs τ_t , and the number of available discrete positions for tRNAs in the cell volume N_t:

$$c_j = \frac{T^f_{\varphi(j)} w_j s}{\tau_t N_t} \qquad (13)$$

To adapt the algorithm for stress conditions, we replaced the tRNA Gene Copy Number (tGCN) used in the algorithm by the adjusted tGCN$_{(s)}$ values that account for the changes in tRNA abundance that we measured in the corresponding stress conditions. We also replaced the mRNA abundance values by $a_{(s)}$ values, as described before. We assumed no changes in ribosome concentration or initiation probability of mRNA transcripts during stress conditions.

The simulation provides various outputs. In particular, translation events are monitored in each gene of the simulated transcriptome during the simulation. Specifically, we analyzed the average waiting-time between consecutive initiation events, $\mu_{\Delta i}$, and the average elongation time over the duration of the simulation, $\mu_{\tau e}$. In addition to these metrics, we introduced additional outputs: (i) translation speed (τ) and (ii) global protein production rates (Π). For each gene, translation speed τ was defined as the number of proteins produced per transcript per unit of time.

$$\tau(g) = \frac{1}{\mu_{\Delta_i}} \times \frac{n_{codons}}{\mu_{\tau_e}}(g) \qquad (14)$$

where $1/\mu_{\Delta i}$ gives the initiation frequency and $n_{codons}/\mu_{\tau e}$ the elongation speed.

For each gene, the protein production rate fold change Π was defined as the quantity of proteins synthesized by the total number of transcripts per unit of time, computed as:

$$\Pi^{(s)}(g) = \frac{a^s}{a^n}(g) \times \frac{\tau^{(n)}(g)}{\tau^{(s)}(g)} \qquad (15)$$

where $a^s/a^n(g)$ is the mRNA fold change of gene g in condition s.

Identification of genes that are better or less adapted or do not show adaptation to stress conditions

To identify the genes that were better adapted (orange) or less adapted (red) or did not show adaptation (grey) to the experimentally measured tRNA abundances, we first computed the rank of the ORF/gene (among all genes) after sorting them based on their n-tAI and s-tAI value. Then, for each sample (stress condition, time point), we:

1. Computed the shift in rank between normal and stress conditions shift(ORFi) = (rank[s-tAI](ORFi) - rank[n-tAI](ORFi))
2. Computed the Z-score of the shift in the sample: Z(ORFi) = (shift(ORFi) - mean(shift)) / sd(shift)
3. Defined the grouping of genes (**Fig. 3A**) based on Z scores: genes whose relative tAI dropped (Z < -1.28, which represents ~10% of genes with highest drop in tAI, red), genes whose relative tAI increased (Z >1.28, which represents ~10% of genes with highest increase in tAI, orange) and other genes (grey).

Bayesian Network analysis

A Bayesian Network (BN) learning approach was used to quantify the conditional dependency of variables related to protein expression: changes in mRNA abundance, tAI, initiation frequency, elongation speed, and finally changes in protein production rate. A BN is formed by: (i) a directed acyclic graph, in which nodes represent variables and edges represent conditional dependencies between variables and (ii) a set of conditional probabilities for each node, which describes its probability distribution given that of its parent-nodes. The global and local distributions are multinomial and are represented as probability or contingency tables.

Nodes in the BN represented the variables enumerated above. To make the variables comparable across stress conditions, each distribution was transformed into deciles (thereby forming 10 equal-sized bins). For instance, in the case of changes in mRNA abundance, the lowest decile represented genes with the strongest decreases in mRNA abundance and the highest decile represented genes with the strongest increase. Measurements from all stress conditions where then gathered, yielding a total of 29,816 observations.

The structure of the BN (or the pattern of connections between the nodes) was computed with the bnlearn R library, using the Incremental Association (IAMB) learning algorithm. The IAMB method consists of two stages, a forward phase (FP) and a backward phase (BP). In the FP, all variables enter the model. In the BP, the false positives are identified and removed. For more details on how the IAMB model works please see https://pdfs.semanticscholar.org/ad36/ee1fc35c48af52ca7bc4d222b2deb6a95409.pdf.

To address the directionality of edges in the BN, a set of forbidden directed edges was defined to exclude models that were impossible theoretically. The Jonckheere-Terpstra test was used to test the conditional independence of variables. The effect size of the relationship between the variables was computed using the "magnitude of impact", which measures the link strength between pairs of variables in the inferred Bayesian Network (as described in the research report here: https://smartech.gatech.edu/handle/1853/29804).

Gene Ontology enrichment tests

Gene Ontology (GO) analyses were done using yeast GO Slims taken from the Saccharomyces Genome Database(*67*). We used a Fisher's exact test to quantify the enrichment or the depletion of GOs in sets of genes of interest. *p*-values were adjusted with the FDR method (**Data File S4**). Orange (better adapted), grey (no change in adaptation) and red (less well adapted) gene sets were defined as those that doubled, remained within 2 fold or halved their rank upon ordering by their s-tAI compared to the n-tAI.

Statistical analyses

Many variables studied are typically not normally distributed, and do not satisfy the requirements for parametric tests (such as the *t*-test) to be applied. When relevant, we thus used the Mann-Whitney U test for comparing pairs of independent samples. Where required, *p*-values were corrected for multiple testing using the FDR correction. The effect sizes of $\chi 2$ tests were computed as: $\Phi=\sqrt{(\chi^2/n)}$ (with n being the sample size).

For the non-parametric comparison of distributions, the Mann-Whitney U test was used. Rank-biserial correlation coefficients were computed to measure effect sizes, with the use of the Wendt formula:

$$r = 1 - \frac{2U}{n_1 n_2} \qquad (16)$$

where U is the Mann-Whitney statistic, and n_1 and n_2 the sizes of the samples being compared. The correlation r expresses the difference between the proportions of pairs that support the hypothesis, minus the proportion of pairs that do not.

Dimensionality reduction using t-SNE and k-means

We used t-SNE, which is a machine learning algorithm that is particularly useful to visualize high-dimensional data (*28*). Its core principle is to attribute each observation (here a tRNA) a particular location in a two (or three) dimensional map, in such a way that similar data points stay nearby each other, and stay away from dissimilar data points. The t-SNE dimension reduction was performed on a dataset consisting of all 12 measurements (four conditions and three time points) for all elongator tRNAs except tRNALeu(CAA), tRNALeu(UAG) and tRNAMet(CAU), because these tRNAs appeared to have a disproportionate influence on scaling. tSNE was used on a Euclidian distance matrix M of the standardized fold changes (namely, $\mu = 0$ and $\sigma^2 = 1$). The perplexity parameter (optimal number of neighbors) was set to five, which is recommended for the sample size of the dataset. To perform the clustering, results from the t-SNE analysis were used as the input to the k-means clustering method. Three dimensions were computed, which also corresponded to the number of significant components found in a PCA performed on the same dataset.

Quantification of protein fold change from confocal microscopy data in oxidative stress

Protein abundance data for yeast in oxidative stress was obtained from Breker *et al.* (*33*). To control for the effect of mRNA abundance on protein expression, the genes were first divided (tertiles) into those with low, medium and high mRNA abundance. For each category, fold change in protein abundance (log2 of the ratio of protein abundance in oxidative stress to normal condition) was plotted for the different groups of genes.

Code availability

All codes are custom written and can be obtained from the authors upon request.

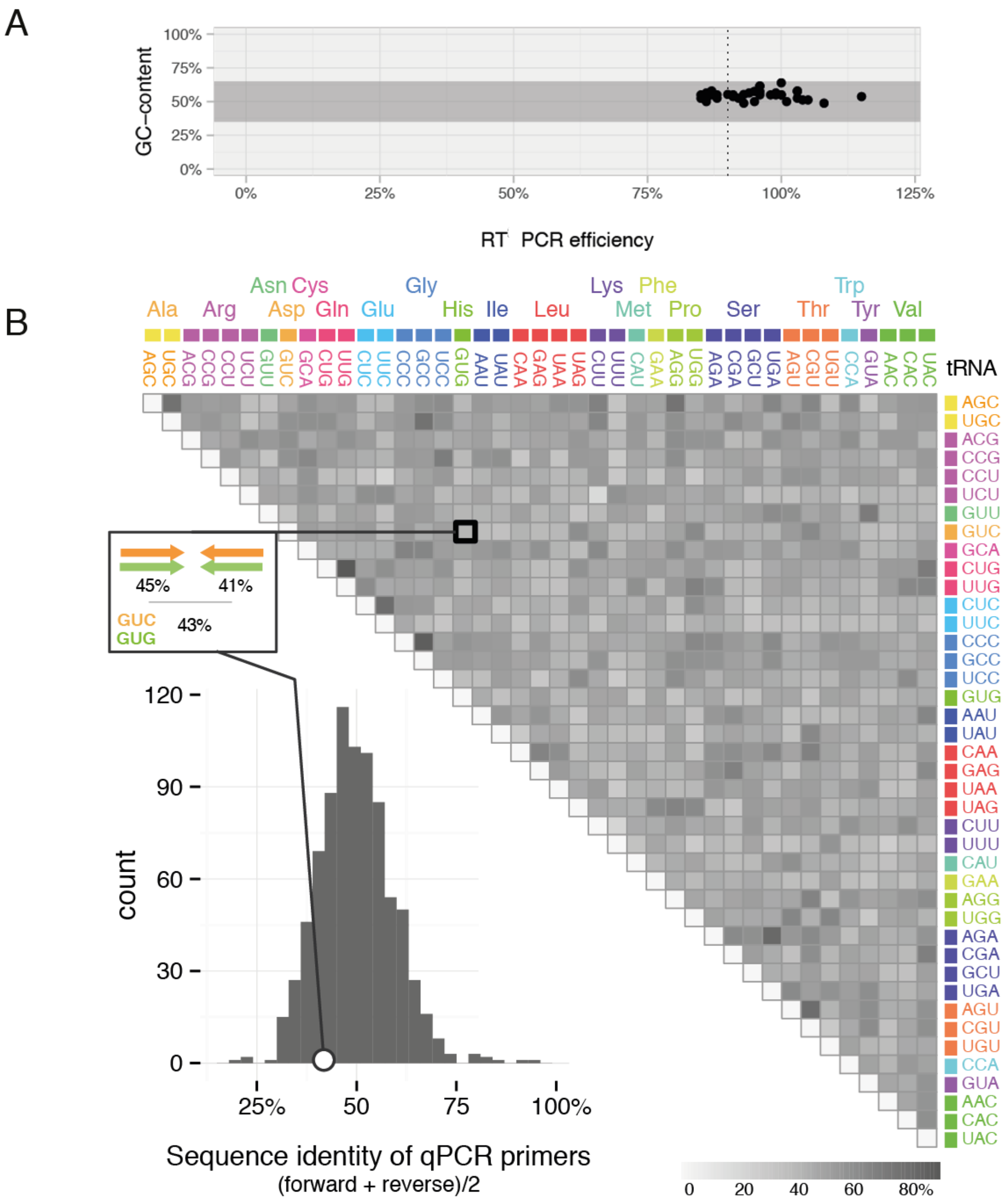

Fig. S1. PCR efficiency, GC content, and sequence identity of PCR primers. (**A**) PCR efficiency was not correlated with the GC content of the primers used. (**B**) Primer sequence identity was defined as the number of identical positions divided by the total number of positions (aligned + internal gaps). Forward and reverse primers were aligned and the average of the two scores is indicated as a shade of grey. An example is given for GUC and GUG, whose qPCR primers have an overall sequence identity of 43%. 812 out of 820 pairs of tRNAs (99%) used primers with less than 75% sequence identity. Pairwise comparison of the sequences of the primers used in the qPCR experiments. A cell (i, j) in the matrix indicates the overall sequence identity of the qPCR primers of $tRNA_i$ and $tRNA_j$. There is no apparent bias in tRNA abundance changes due to primer sequence identity. The diagonal elements are intentionally colored white.

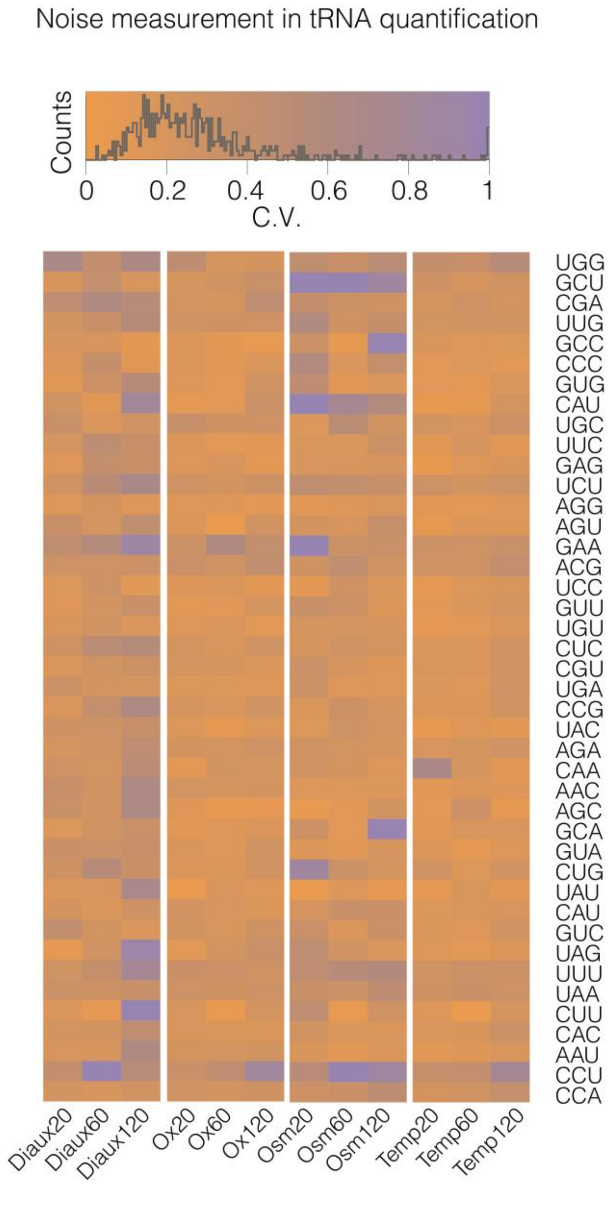

Fig. S2. Noise associated with each tRNA measurement for the different stress conditions. Noise was measured as σ/μ.

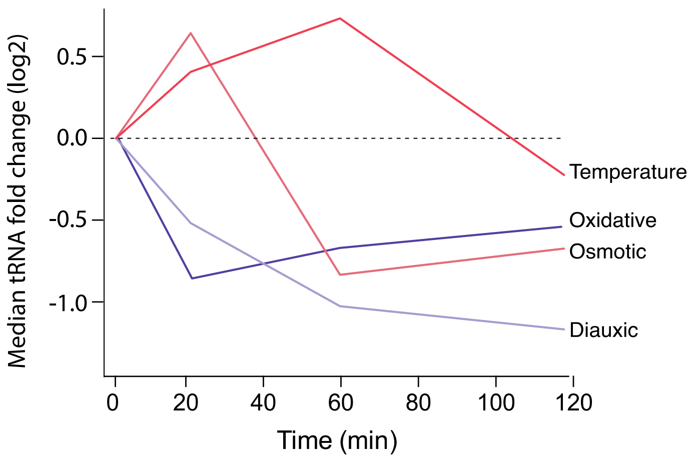

Fig. S3. Biphasic response of tRNA abundance change upon exposure to stress. Relative changes in tRNA abundance change over time. The x-axis shows time after stress induction and the y-axis shows the median value of the measured change in the abundance of the 42 different tRNAs (on a $\log_2$ scale, 0 indicating no change).

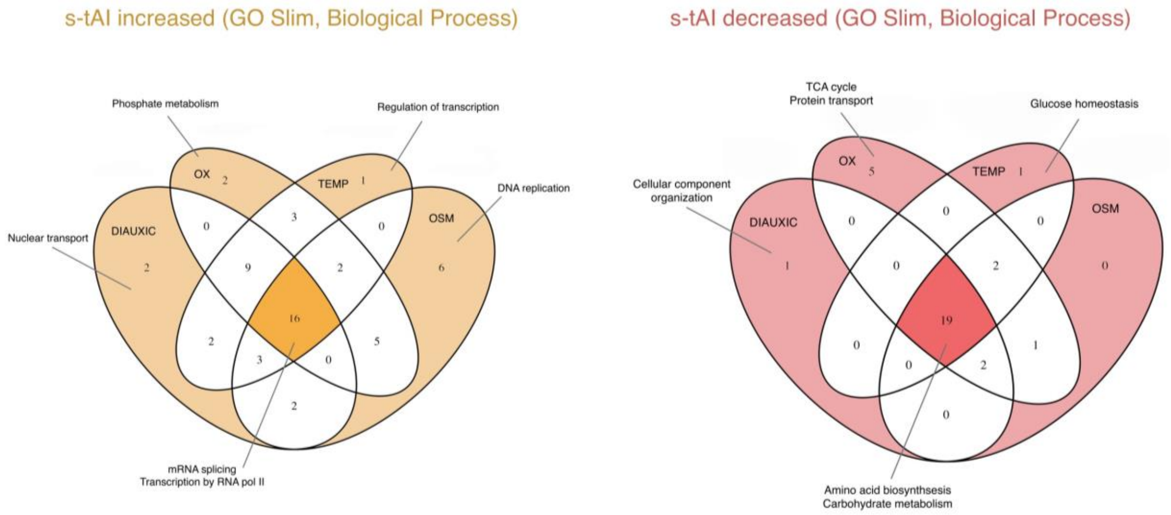

Fig. S4. Functional enrichment of genes whose codons are better or less well adapted to stress conditions. Enrichment of GO slim annotations for genes with significant differences in the tAI between normal and stress conditions (see **Fig. 3A** for the definition of the two groups of genes). P-values for GO slim enrichment can be found in **Data File S4**.

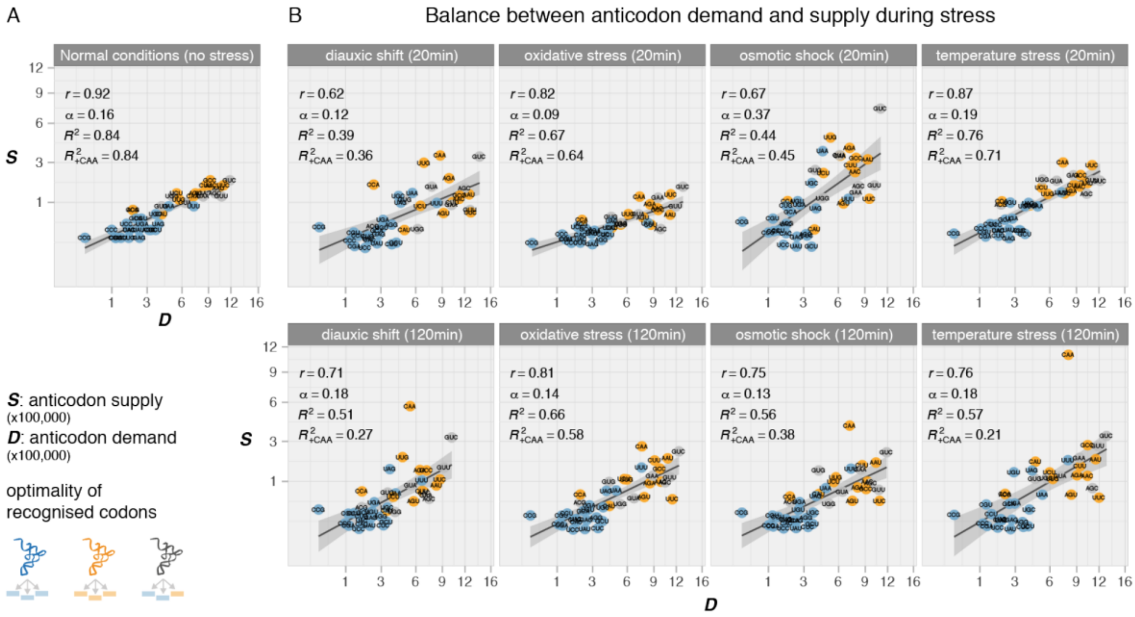

Fig. S5. Balance between anticodon supply and demand in stress conditions. (**A**) Normal condition and (**B**) stress conditions. Anticodon demand corresponds to the sum of occurrences of individual codons in the studied transcriptome. Anticodons that can adapt wobble codon-anticodon pairs (low affinity, non-optimal) were colored blue; anticodons that can adapt only to Watson & Crick pairs (high affinity, optimal) were colored orange; and those that can adapt both types were colored grey. For more details, see the Materials and Methods in the Supplementary Materials. *r* scores indicate Pearson correlation coefficients. *α* shows the slope of the regression of anticodon supply on demand ($S = \alpha D + \beta$). Coefficient of determination are given in the absence (R^2) or presence of the outlier, $tRNA^{Leu(CAA)}$ (R^2_{+CAA}). Regression lines are shown with 95% confidence intervals. All axes are shown on a square root scale to improve the readability of data points for conditions in which $tRNA^{Leu(CAA)}$ is an outlier.

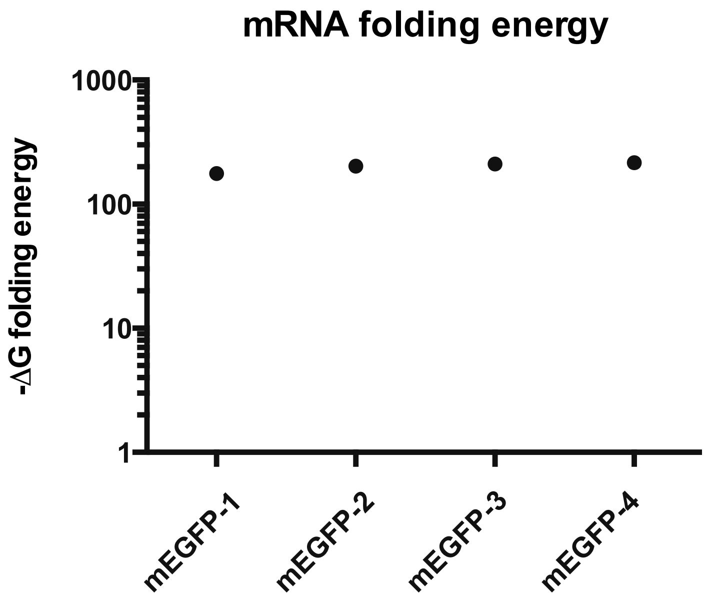

Fig. S6. Computed mRNA folding energy of the four different mEGFP variants. Calculated folding energy (-ΔG) for the four different designed mEGFP variants. The RNAfold server included in the ViennaRNA package (*68*) was used to compute RNA folding energy. See Materials and Methods in the Supplementary Materials.

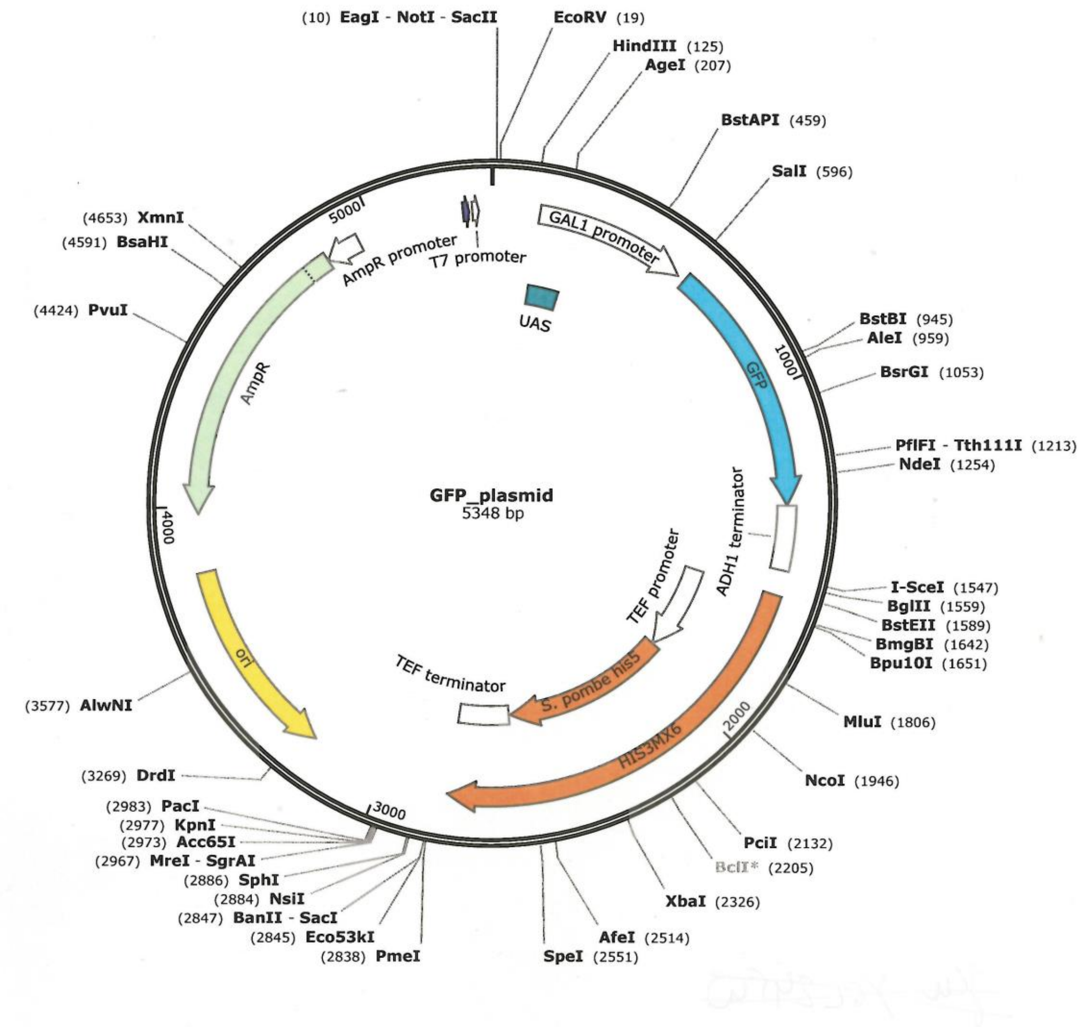

Fig. S7. Plasmid map used for integrating the mEGFP variants into the yeast genome. The pMA plasmid used to integrate the designed mEGFP sequences into the TRP1 locus of the yeast genome. All mEGFP versions were synthesized de novo and inserted between pGal1 and TEF terminator.

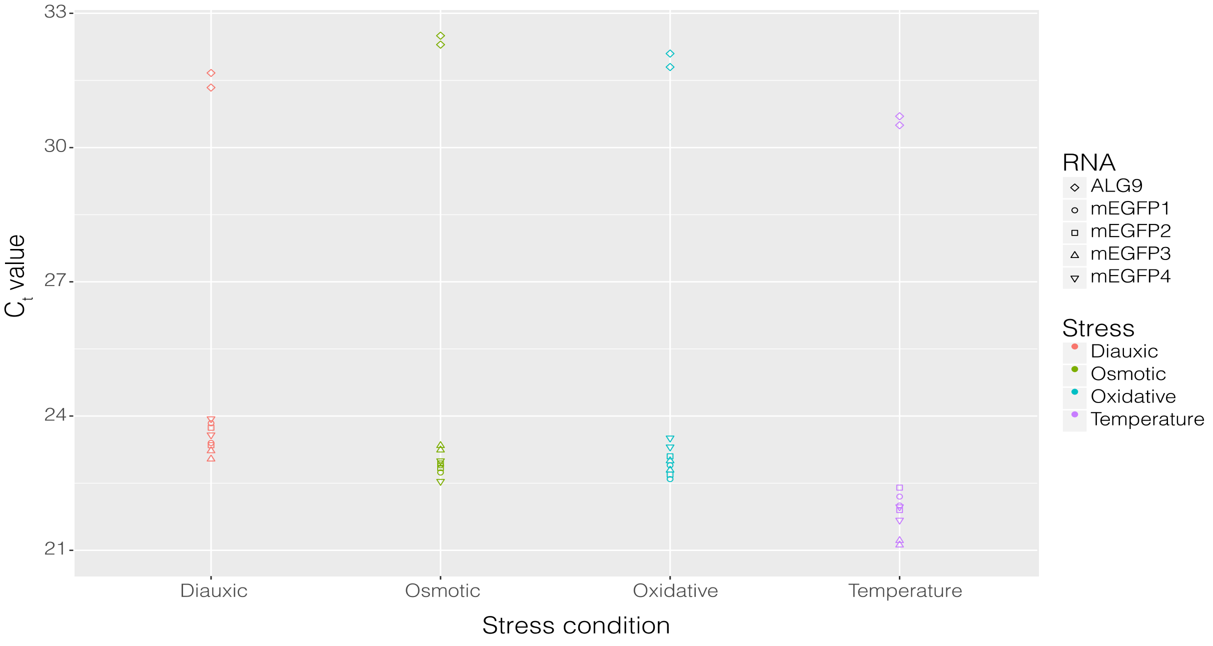

Fig. S8. mRNA abundance measurement for the mEGFP variants under stress conditions. Measured mRNA levels for all mEGFP variants using a RT-qPCR approach. Ct values were measured also for the housekeeping gene ALG9 to control for total RNA. Two replicates were measured in each condition.

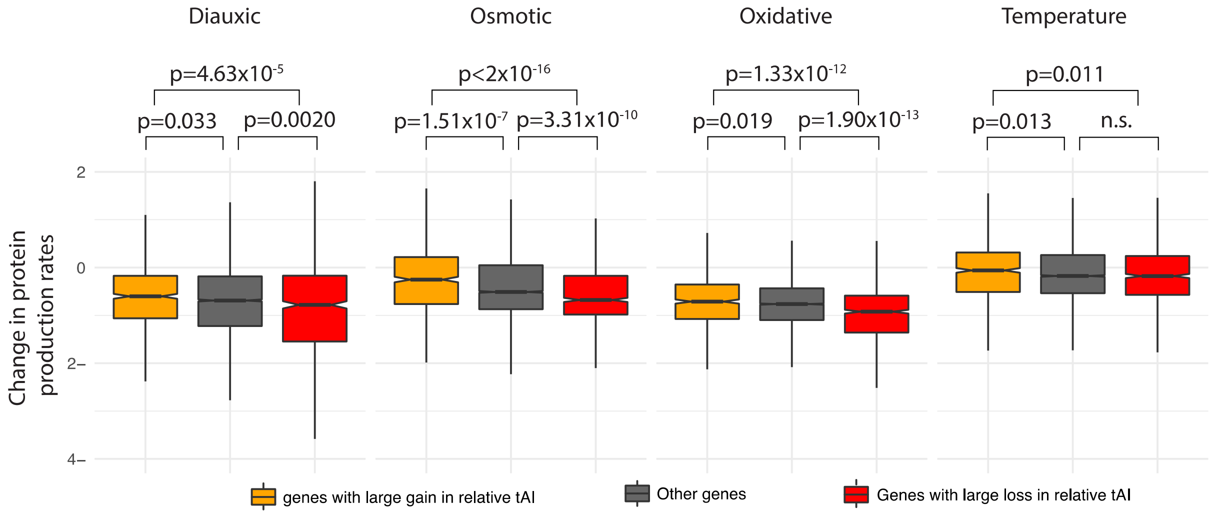

Fig. S9. Stress-induced fold changes in protein production rates during simulation. The groups of genes are those that are better adapted (orange), not affected (grey), and less adapted (red) to the experimentally measured tRNA pool. On a global scale, we found a general tendency for genes that become less adapted to the tRNA pool to show a decrease in protein production rates compared to the genes that are better adapted to the stress tRNA pool (see also **Fig. 5B**). However, the differences were either not significant or of borderline significance for the simulations under temperature stress. Additional regulatory mechanisms such as transcript-specific translation repression and initiation contribute to the protein production in temperature stress (*37, 69*) and these are not explicitly modeled in the simulation. See Materials and Methods in the Supplementary Materials.

Table S1. Oligos used for tRNA quantification.

tRNA Anticodon	Forward primer	Reverse primer
TGG	TGGTATGATTCTCGCTTTGGGT	AGCTGGGAATTGAACCCAGG
GCT	CCAGTGGCCGAGTGGTTAAG	GGTAAAACCCAATGCCTTAGCA
CGA	GGCACTATGGCCGAGTGGTT	CAGAGCCCAAGAGATTTCGAGT
TTG	TGTAGTGGTTATCACTTTCGGT	GGATTCGAACCGGGGTTGT
GCC	GCGCAAGTGGTTTAGTGGTA	GCAAGCCCGGAATCGAAC
CCC	CGCAAGTGGTTCAGTGGTTAG	GCAAGCCGGGAATCGAAC
GTG	TACACATCGTTGTGGCCGAT	TGCCATCTCCTAGAATCGAACC
(initiation)CAT	AGTGGAAGCGCGCAGG	CTCGGTTTCGATCCGAGGAC
TGC	CACATGGCGAGTTGGTAG	CGGAATCGAACCGATGACCT
TTC	ACGGCTATCACATCACGCTT	CTCCGATACGGGGAGTCGAA
GAG	ACTCTGGCCGAGTGGTCTAA	GCCTCCGAAGAGATCAGGAC
TCT	GGCAACGCGTCTGACTTCTA	ACTCACGATGGGGGTCGAA
AGG	GGCGTGTGGTCTAGAGGTATG	ACTCGAACCCGGGACCTC
AGT	CAAGTTGGTAAGGCGCCAC	TCGGATTTGAACCGATGATCTCC
GAA	GAGAGCGCCAGACTGAAGAT	TGCGAACTCTGTGGATCGAA
ACG	AATGGTCACGGCGTCTGG	GCCAGGACTTGAACCTGGAA
TCC	GGGCGGTTAGTGTAGTGGTT	ACGAGAATCGAACCCGTGTC
GTT	CCAAGTTGGTTAAGGCGTGC	ACCCCAGTGAGGGTTGAACT
TGT	GTGGTAGAGCGTTGCACTTG	GCCACCTGCCAGAATTGAAC
CTC	ACGGCTATCACATCACGTTCT	CGAAGCGGGGAGTCGAAC
CGT	TTTGGCCAAGTGGTAAGGCA	AATTGAACCCACGATCCCCG
TGA	CGAGTGGTTAAGGCGACAGA	ACACCAGCAGGATTTGAACCA
CCG	GCTCCTCTAGTGCAATGGTT	GACTCGAACCCGGATCACAG
TAC	TGGTCCAGTGGTTCAAGACG	TTCGAACTCGGGATCTTCGC
AGA	ACTTGGCCGAGTGGTTAAGG	ACAACTGCAGGACTCGAACC
CAA	TTTGGCCGAGCGGTCTAAGG	TGCATCTTACGATACCTGAGCTT
AAC	GGTTTCGTGGTCTAGTCGGT	GGACGTTCTGCGTGTTAAGC
AGC	CGTGTGGCGTAGTCGGTAG	CGGAATTCGAACCGGAGACC
GCA	CTCGTATGGCGCAGTGGTAG	CTCGCACTCAGGATCGAACT
GTA	AGCCAAGTTGGTTTAAGGCG	GAACGCCCGATCTCAAGATTTAC
CTG	AGTGGTTATCACTTTCGGTTCTGA	CCGGATTCGAACTGGGGTTG
TAT	GCTCGTGTAGCTCAGTGGTT	CCACGACGGTCGCGTTAT
(elongation)CAT	GCTCAGTAGGAAGAGCGTCA	GGTTCGAACTCTCGACCTTCA
GTC	AATGGTCAGAATGGGCGCTT	GGGGAATTGAACCCCGATCT
TAG	AGGCGTCAGATTTAGGCTCTG	GGGATTCGAACCCTTGCATC
TTT	AGAGCGTTCGGCTTTTAACC	CTCCTCATAGGGGGCTCGAA
TAA	GTTGGCCGAGTGGTCTAAGG	CGGACAACCGTCCAACAGAT
CTT	CTTGTTGGCGCAATCGGTAG	GGGCTCGAACCCCTAACCTT
CAC	TGTAGCGGCTATCACGTTGC	AGGATCGAACTCGGGACCTT
AAT	GTCTCTTGGCCCAGTTGGTT	TGGTCTCTAGCGGGATCGAA
CCT	TCCGTTGGCGTAATGGTAAC	GAACCCGCAGTCTTCTCCTT
CCA	GAAGCGGTGGCTCAATGGTA	CGGACAGGAATTGAACCTGC

Table S2. mEGFP variant sequences.

>mEGFP-1	ATGGTCTCTAAAGGTGAGGAGTTGTTTACGGGCGTTGTTCCCATTTTGGTCGAGTTGGACGGCGA<br>TGTCAATGGTCACAAATTCTCCGTTTCCGGCGAGGGCGAGGGCGATGCAACGTACGGTAAATTGA<br>CGTTGAAATTTATTTGCACGACGGGTAAATTGCCTGTTCCTTGGCCTACGTTGGTTACGACGTTG<br>ACGTACGGCGTTCAATGTTTCTCTCGGTATCCCGACCACATGAAACAACACGATTTCTTTAAATC<br>CGCGATGCCTGAGGGTTATGTTCAAGAGCGGACGATCTTTTTCAAAGATGACGGCAATTACAAAA<br>CGCGGGCGGAGGTCAAATTCGAGGGTGACACGTTGGTTAACCGGATCGAGTTGAAAGGCATTGAT<br>TTTAAAGAGGATGGCAACATTTTGGGCCACAAATTGGAGTATAACTATAATTCCCATAATGTCTA<br>CATCATGGCAGATAAACAAAAAAATGGTATCAAAGTCAATTTCAAAATCCGGCACAATATTGAGG<br>ATGGTTCTGTTCAATTGGCAGATCACTATCAACAAAATACGCCTATCGGTGATGGCCCTGTCTTG<br>TTGCCCGACAATCACTATTTGTCCACGCAATCCAAATTGTCCAAAGACCCCAATGAGAAACGGGA<br>TCATATGGTCTTGTTGGAGTTCGTTACGGCGGCGGGTATTACGTTGGGTATGGATGAGTTGTATA<br>AATAA
>mEGFP-2	ATGGTGTCGAAAGGAGAAGAACTGTTTACGGGAGTGGTGCCCATACTGGTGGAACTAGACGGAGA<br>TGTGAATGGACACAAATTCTCGGTGTCGGGAGAAGGAGAAGGAGATGCAACGTACGGAAAACTAA<br>CGCTAAAATTTATATGCACGACGGGAAAACTACCTGTGCCTTGGCCTACGCTAGTGACGACGCTG<br>ACGTACGGAGTGCAGTGTTTCTCGAGGTATCCCGACCACATGAAACAGCACGATTTCTTTAAATC<br>GGCGATGCCTGAAGGATATGTGCAGGAAAGGACGATATTTTTCAAAGATGACGGAAATTACAAAA<br>CGAGGGCGGAAGTGAAATTCGAAGGAGACACGCTGGTGAACAGGATAGAACTAAAAGGAATAGAT<br>TTTAAAGAAGATGGAAACATACTAGGACACAAACTAGAATATAACTATAATTCGCATAATGTGTA<br>CATAATGGCAGATAAACAGAAAAATGGAATAAAAGTGAATTTCAAAATAAGGCACAATATAGAAG<br>ATGGATCGGTGCAGCTAGCAGATCACTATCAGCAGAATACGCCTATAGGAGATGGACCTGTGCTG<br>CTACCCGACAATCACTATCTATCGACGCAGTCGAAACTATCGAAAGACCCCAATGAAAAAAGGGA<br>TCATATGGTGCTACTGGAATTCGTGACGGCGGCGGGAATAACGCTGGGAATGGATGAACTATATA<br>AATAA
>mEGFP-3	ATGGTATCTAAGGGCGAGGAGTTGTTTACGGGCGTAGTACCAATCTTGGTAGAGTTGGACGGTGA<br>CGTAAATGGTCATAAGTTTTCTGTATCTGGTGAGGGCGAGGGTGACGCGACGTATGGCAAGTTGA<br>CGTTGAAGTTTATTTGTACGACGGGTAAGTTGCCAGTACCGTGGCCAACGTTGGTAACGACGTTG<br>ACGTACGGTGTACAGTGCTTCTCCCGGTACCCAGACCATATGAAGCAGCATGACTTCTTTAAGTC<br>CGCAATGCCAGAGGGCTACGTACAGGAGCGGACGATCTTCTTCAAGGACGACGGCAATTATAAGA<br>CGCGGGCGGAGGTAAAGTTTGAGGGCGACACGTTGGTAAACCGGATCGAGTTGAAGGGCATCGAT<br>TTCAAGGAGGATGGCAATATTTTGGGCCACAAGTTGGAGTATAATTATAATTCCCATAATGTATA<br>TATTATGGCAGATAAGCAGAAGAATGGTATCAAGGTAAACTTTAAGATTCGGCATAACATTGAGG<br>ACGGTTCTGTACAGTTGGCGGATCATTATCAGCAGAATACGCCGATCGGCGACGGCCCGGTATTG<br>TTGCCAGACAATCATTACTTGTCCACGCAGTCTAAGTTGTCTAAGGACCCGAATGAGAAGCGGGA<br>TCACATGGTATTGTTGGAGTTCGTAACGGCGGCAGGTATCACGTTGGGCATGGACGAGTTGTACA<br>AGTAA
>mEGFP-4	ATGGTTTCCAAAGGCGAGGAGCTGTTTACAGGTGTTGTTCCAATTCTGGTCGAGCTGGACGGCGA<br>TGTTAATGGTCATAAATTCTCCGTCTCCGGCGAGGGCGAGGGCGATGCGACATATGGTAAACTAA<br>CACTAAAATTTATTTGCACAACAGGCAAACTACCGGTTCCATGGCCAACACTGGTCACAACACTA<br>ACATACGGTGTCCAGTGTTTTTCTAGGTACCCAGACCACATGAAACAGCACGACTTCTTTAAATC<br>TGCAATGCCGGAGGGCTATGTTCAGGAGAGGACAATTTTCTTTAAAGACGATGGCAATTACAAAA<br>CAAGGGCGGAGGTTAAATTTGAGGGTGATACACTGGTTAATAGGATCGAGCTAAAAGGCATTGAC<br>TTTAAAGAGGACGGCAACATTCTAGGCCATAAACTAGAGTATAACTATAATTCTCACAATGTCTA<br>CATTATGGCGGACAAACAGAAAAACGGCATCAAAGTCAACTTTAAAATTAGGCATAACATTGAGG<br>ACGGCTCTGTCCAGCTAGCGGACCACTATCAGCAGAACACACCAATTGGTGATGGCCCGGTCCTG<br>CTGCCGGATAATCACTATCTGTCTACACAGTCTAAACTATCTAAAGACCCGAACGAGAAAAGGGA<br>CCATATGGTCCTGCTGGAGTTTGTTACAGCGGCGGGCATCACACTAGGCATGGATGAGCTATACA<br>AATAA

Table S3. Oligos used for mRNA quantification.

GFP sequence	Forward primer	Reverse primer
mEGFP-1	ATGGCAACATTTTGGGCCAC	GACAGGGCCATCACCGATAG
mEGFP-2	GAAGGAGACACGCTGGTGAA	GATCTGCTAGCTGCACCGAT
mEGFP-3	TTTGGGCCACAAGTTGGAGT	TCCGCCAACTGTACAGAACC
mEGFP-4	GGGCGATGCGACATATGGTA	GTGTTGTGACCAGTGTTGGC
